# Specificities of Human Hepatocellular Carcinoma Developed on Non-Alcoholic Fatty Liver Disease in Absence of Cirrhosis Revealed by Tissue Extracts ^1^H-NMR Spectroscopy

**DOI:** 10.3390/metabo7040049

**Published:** 2017-09-22

**Authors:** Camille Teilhet, Daniel Morvan, Juliette Joubert-Zakeyh, Anne-Sophie Biesse, Bruno Pereira, Sylvie Massoulier, Pierre Dechelotte, Denis Pezet, Emmanuel Buc, Géraldine Lamblin, Michel Peoc’h, Jack Porcheron, Marie-Paule Vasson, Armando Abergel, Aicha Demidem

**Affiliations:** 1 Department of Digestive and Hepatobiliary Medecine, University Medical Hospital, F-63000 Clermont-Ferrand, France; cteilhet@chu-clermontferrand.fr (C.T.); smassoulier@chu-clermontferrand.fr (S.M.); glamblin@chu-clermontferrand.fr (G.L.); aabergel@chu-clermontferrand.fr (A.A.); 2 INRA, Human Nutrition Unit, CRNH Auvergne, Clermont Auvergne University, F-63000 Clermont-Ferrand, France; m-paule.vasson@uca.fr; 3 Laboratory of Biophysics and Image Processing, Clermont Auvergne University, F-63000 Clermont-Ferrand, France; daniel.morvan@uca.fr; 4 Department of Anatomo-pathology, University Medical Hospital, F-63000 Clermont-Ferrand, France; jjoubert@chu-clermontferrand.fr (J.J.-Z.); pdechelotte@chu-clermontferrand.fr (P.D.); 5 Team RMN-START, Clermont Auvergne University, F-63000 Clermont-Ferrand, France; a-sophie.biesse-martin@uca.fr; 6 Department of Clinical Research & Innovation, University Medical Hospital, F-63000 Clermont-Ferrand, France; bpereira@chu-clermontferrand.fr; 7 Department of Digestive Surgery, University Medical Hospital, F-63000 Clermont-Ferrand, France; dpezet@chu-clermontferrand.fr (D.P.); ebuc@chu-clermontferrand.fr (E.B.); 8 Department of Anatomo-pathology, University Medical Hospital, F-42000 Saint-Etienne, France; michel.peoch@chu-st-etienne.fr; 9 Department of Digestive Surgery, University Medical Hospital, F-42000 Saint-Etienne, France; jack.porcheron@chu-st-etienne.fr; 10 UMR CNRS 6284, Clermont Auvergne University, F-63000 Clermont-Ferrand, France

**Keywords:** hepato-cellular carcinoma, ^1^H-NMR spectroscopy, metabolomics, cirrhosis, NAFLD, metabolites, biomarkers, glutamine synthetase

## Abstract

There is a rising incidence of non-alcoholic fatty liver disease (NAFLD) as well as of the frequency of Hepato-Cellular Carcinoma (HCC) associated with NAFLD. To seek for putative metabolic pathways specific of the NAFLD etiology, we performed comparative metabolomics between HCC associated with NAFLD and HCC associated with cirrhosis. The study included 28 pairs of HCC tissue versus distant Non-Tumoral Tissue (NTT) collected from patients undergoing hepatectomy. HCC was associated with cirrhosis (*n* = 9), normal liver (*n* = 6) and NAFLD (*n* = 13). Metabolomics was performed using 1H-NMR Spectroscopy on tissue extracts and combined to multivariate statistical analysis. In HCC compared to NTT, statistical models showed high levels of lactate and phosphocholine, and low level of glucose. Shared and Unique Structures (SUS) plots were performed to remove the impact of underlying disease on the metabolic profile of HCC. HCC-cirrhosis was characterized by high levels of β-hydroxybutyrate, tyrosine, phenylalanine and histidine whereas HCC-NAFLD was characterized by high levels of glutamine/glutamate. In addition, the overexpression glutamine/glutamate on HCC-NAFLD was confirmed by both Glutamine Synthetase (GS) immuno-staining and NMR-spectroscopy glutamine quantification. This study provides evidence of metabolic specificities of HCC associated with non-cirrhotic NAFLD versus HCC associated with cirrhosis. These alterations could suggest activation of glutamine synthetase pathway in HCC-NAFLD and mitochondrial dysfunction in HCC-cirrhosis, that may be part of specific carcinogenic processes.

## 1. Introduction

Liver cancer is the sixth most frequently diagnosed cancer worldwide, and the second leading cause of cancer death [1]. Hepatocellular Carcinoma (HCC) mostly develops in patients with cirrhosis, a well-known precancerous condition.

Since the past 20 years, the increasing incidence of HCC is suspected to be related to the increasing burden of Non-Alcoholic Fatty Liver Disease (NAFLD) [2,3]. NAFLD is closely linked to obesity, type 2 diabetes, dyslipidemia and insulin resistance, and considered as the liver manifestation of the metabolic syndrome [4]. NAFLD refers to a body of liver diseases including simple steatosis, steato-hepatitis, fibrosis and cirrhosis. NAFLD has become the most common liver disorder in industrialized countries, affecting up to 20% of the adult population in western countries.

Several studies report a high proportion (up to 40%) of non-cirrhotic NAFLD, as the underlying liver disease, in patients with HCC, at odds with the paradigm that cirrhosis is a necessary step in carcinogenesis [2,5,6,7]. Indeed, recent epidemiological data suggest that carcinogenesis might occur in NAFLD without being mediated by fibrosis [5].

Most of NAFLD patients are asymptomatic and early detection of HCC in this context is a major challenge. More investigation is needed to identify pro-carcinogenic factors in NAFLD without fibrosis.

The putative pathways linking steatosis and steato-hepatitis to HCC have been little investigated. Potential carcinogenic mechanisms include inflammation with increased release of tumor necrosis factors (TNF)-alpha and interleukin 6, altered release of adipokines through insulin resistance and inflammation, lipotoxicity inducing oxidative stress and DNA damage [8,9].

Metabolomics is being widely used to gain insights into carcinogenesis mechanisms. Most metabolomics studies of HCC have been applied to serum and urine of patients, using Mass Spectrometry or Nuclear Magnetic Resonance (NMR) spectroscopy [10,11,12]. At present, no metabolomics study has investigated HCC histologically proved to be associated to NAFLD.

The aim of our study is to propose a specific metabolic profile of HCC depending of the underlying disease: cirrhosis versus NAFLD.

In this study, we performed ^1^H-NMR spectroscopy-based metabolomics of HCC tissue in patients with non-cirrhotic NAFLD or cirrhosis, to seek for specific features of HCC in both underlying diseases.

We show that HCC associated with cirrhosis specifically exhibits high levels of β-hydroxybutyrate (β-HB), tyrosine (Tyr) and phenylalanine (Phe), histidine (His) whereas HCC associated with non-cirrhotic NAFLD specifically exhibits high level of glutamine/glutamate. This data is correlated with immunohistochemical expression of Glutamine Synthetase. All together, these observations may reflect specific carcinogenic processes.

## 2. Results

### 2.1. Patients

Our HCC cohort (*n* = 28) included 23 males and 5 females with a mean age of 69 years. Among the 28 HCC, 9 were associated with cirrhosis (HCC-cirrhosis), and 19 associated with non-cirrhotic liver tissue (HCC-Non cirrhosis). Histological examination indicated that among the 19 patients with HCC-Non-cirrhosis, 6 had a normal Non Tumoral Tissue (NTT) and 13 had NAFLD (HCC-NAFLD), including 7 steatosis and 6 Non Alcoholic Steato-Hepatitis (NASH). Clinical, biological, histological features of the 2 groups (HCC-Cirrhosis and HCC-NAFLD) are reported in the Table 1 Serum AFP level <20 ng/mL was found in 85% of patients with HCC-NAFLD versus 45% in patients with HCC-cirrhosis (*p* = 0.047).

### 2.2. Metabolomics Comparison of HCC to NTT

**Figure 1:**
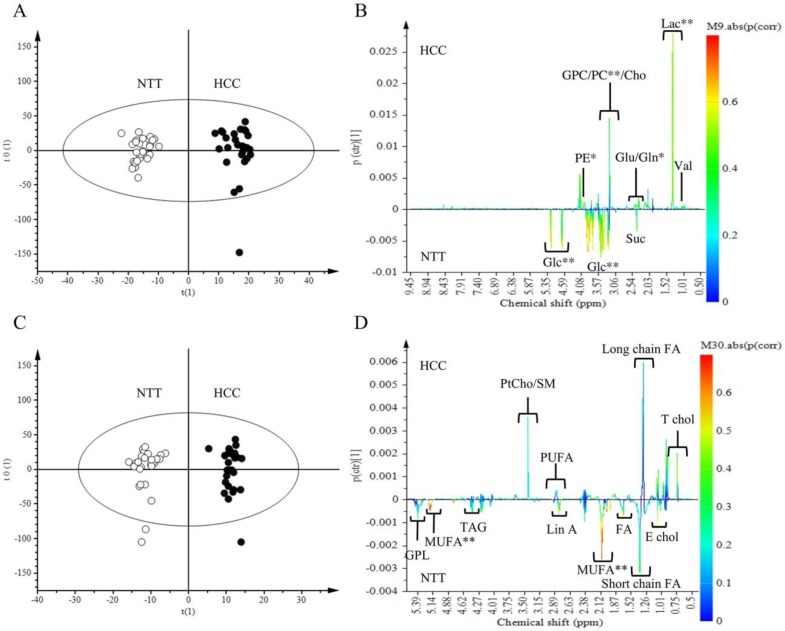
Discrimination of Hepato-Cellular Carcinoma (HCC) tissue from Non-Tumoral Tissue (NTT): aqueous and lipid metabolites analysis. Orthogonal Partial Least Square-Discriminant Analysis (OPLS-DA) score scatter plot and loading S-line plot of HCC versus NTT from aqueous extract data (**A**,**B**) and lipid extract data (**C**,**D**). On the score plots, each dot corresponds to a spectrum colored according to histology (black for HCC; White for NTT). The constructed model displays a good separation between HCC and NTT. On the loading plot, variations of bucket intensities are represented from 0 to 9 ppm for aqueous extract data and from 0 to 6 ppm for lipid extract data. Positive signals correspond to the metabolites present at higher concentrations in HCC. While negative signals represent the metabolites present at higher levels in NTT. The first model (**A**,**B**) was built with 1 predictive and 1 Y-orthogonal components and exhibited an explained variance: (R2X) of 0.61, (R2Y) of 0.53, predictability (Q2Y) of 0.40. The second model (**C**,**D**) was built with 1 predictive and 6 Y-orthogonal components and exhibited an explained variance: (R2X) of 0.55, (R2Y) of 0.97, predictability (Q2Y) of 0.50. The buckets are displayed according to the colored scale of correlation coefficient *p* (corr) (**: *p* (corr) > |0.7|; *: |0.5| < *p* (corr) < |0.7|).

We compared the overall metabolic profile of HCC to NTT from aqueous and lipid extracts. Tissue spectra of HCC and NTT groups were separated by OPLS-DA with aqueous extract data and lipid extract data (Figure 1A,C respectively). Multivariate analysis showed that HCC tissue is characterized by high level of lactate (Lac) (*p* (corr) > 0.7), phosphocholine (PC), phosphoethanolamine (PE), glutamine (Gln) (*p* (corr) > 0.5) and low level of glucose (Glc) and monounsaturated fatty acids (MUFA) (*p* (corr) > 0.7) (Figure 1B,D). Forty-five identified metabolites were quantified from aqueous and lipid extracts, according a technique derived from [13]. Univariate analysis showed that 23 metabolites had a significant variation (Table 2). OPLS-DA was performed with the quantified metabolites (data no shown). As expected, the S-plot confirmed the value of Lac as a biomarker of HCC. Analysis of quantified metabolites has the advantage of applying the same weight to each metabolite. By removing heavily contributive metabolites, such as lactate, the PC became a second biomarker of HCC tissue (data not shown).

### 2.3. Metabolites Quantification

Metabolite signals were quantified in all tissue spectra. For quantitative metabolomics profiling, spectra were imported into MestReNova and assigned signals were integrated. Quantification was performed by relating metabolite signals to a residual protein signal (lysine at 2.99 ppm), according to a technique derived from [13]. The Wilcoxon test was used to compare NTT and tumour tissues. In the comparison of metabolites levels between the groups of HCC tissue, Kruskal–Wallis test with Dunn’s correction was used (XLSTAT.V 2014.5.02, Addinsoft).

### 2.4. Comparison of HCC-NAFLD to HCC-Cirrhosis

**Figure 2:**
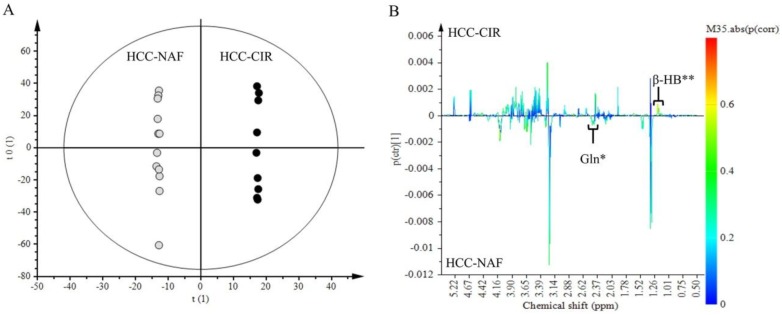
Discrimination of HCC-NAFLD from HCC-Cirrhosis: aqueous metabolites analysis. OPLS-DA discriminated HCC-NAFLD (HCC-NAF) from HCC-cirrhosis (HCC-CIR) with 1 predictive and 5 Y-orthogonal components and exhibited an explained variance: (R2X) of 0.46, (R2Y) of 1, predictability (Q2Y) of 0.30. (**A**) Score plot: each dot corresponds to a spectrum colored according to histology (black for HCC-cirrhosis; Grey for HCC-NAFLD). The constructed model displays a good separation between the spectra of the 2 groups. (**B**) Loading S-line plot: variations of bucket intensities are represented using a line plot from 0 to 9 ppm with a color scale for correlation coefficient. *: |0.5| > *p* (corr) > |0.4|; **: *p* (corr) > |0.5|.

NMR spectra of aqueous extracts of HCC associated with non-cirrhotic NAFLD (*n* = 13) was compared to those of HCC with cirrhosis (*n* = 9) (Figure 2A,B). Lac (1.30–1.35 ppm) and Glc (4.61–4.67 ppm) did not contribute to the discrimination since these signals were common to both HCC groups. Among signals contributing to the discrimination, 2 metabolites were identified: β-HB (1.18 ppm) and Gln (2.45 ppm). β-HB (*p* (corr) = 0.58) was highly expressed in HCC-cirrhosis whereas Gln (*p* (corr) = 0.45) was highly expressed in HCC-NAFLD (Figure 2B).

**Figure 3:**
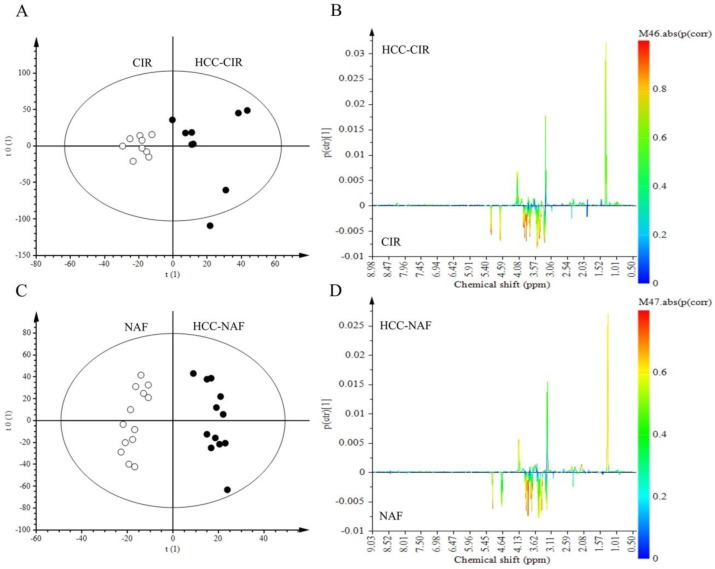
Discrimination of HCC-NAFLD and HCC-Cirrhosis from their own NTT: aqueous metabolites analysis. (**A**,**B**) OPLS-DA discriminated HCC-CIR from Cirrhosis tissue (CIR) with 1 predictive and 1 Y-orthogonal components and exhibited an explained variance: (R2X) of 0.44, predictability (Q2Y) of 0.18. (**C**,**D**) OPLS-DA discriminated HCC-NAF from NAFLD tissue (NAFLD) with 1 predictive and 2 Y-orthogonal components and exhibited an explained variance: (R2X) of 0.35, predictability (Q2Y) of 0.42.

Shared and Unique Structures (SUS) plots were performed to remove the impact of underlying disease on the metabolic profile of HCC and thus, to reveal specific features of HCC-NAFLD and HCC-cirrhosis. OPLS-DA models were obtained for HCC-cirrhosis and HCC-NAFLD and compared with a NTT group (cirrhosis or NAFLD tissues). Two models were generated with HCC-NAFLD and HCC-cirrhosis in comparison with their own control groups (NTT cirrhosis (*n* = 9) and NTT NAFLD (*n* = 13)). HCC associated with normal tissue were excluded (*n* = 6). OPLS-DA discriminated HCC-Cirrhosis from Cirrhosis tissue (R2X = 0.44, Q2Y = 0.18) and HCC-NAFLD from NAFLD tissue (R2X = 0.35, Q2Y = 0.42) (Figure 3).

**Figure 4:**
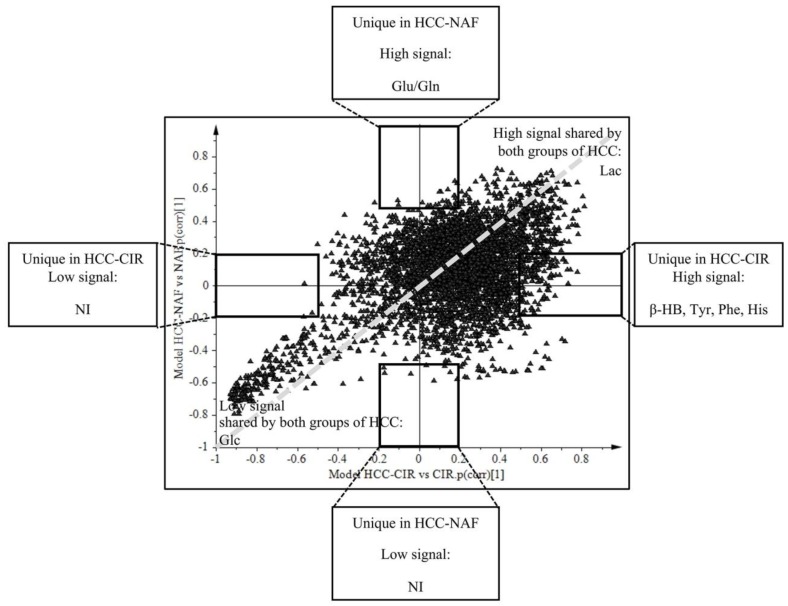
Common and specific metabolites of HCC-cirrhosis and HCC-NAFLD revealed by SUS-plot. Shared and Unique Structures-Plot (SUS-Plot) of 2 models (shown in Figure 3): model A (Orthogonal Partial Least Square-Discriminant Analysis (OPLS-DA) discriminated HCC-cirrhosis (HCC-CIR) from Cirrhosis (CIR), and model C (OPLS-DA discriminated HCC-NAFLD (HCC-NAF) from NAFLD (NAF). HCC-cirrhosis was characterized by increased level β-HB (β-hydroxybutyrate) (signal 1.18 ppm with *p* (corr) > 0.5 and signal 1.19–1.20 ppm with *p* (corr) = 0.45), Tyr (tyrosine) (signals 3.94–3.95 ppm, 6.88–6.91 ppm with *p* (corr) > 0.5 ; signal 7.17–7.18 ppm with *p* (corr) = 0.45), Phe (phenylalanine) (signal 7.34–7.37 ppm with *p* (corr) > 0.5 ; signal 7.40–7.44 ppm with *p* (corr) = 0.45) and His (histidine) (signal 7.07–7.09 ppm with *p* (corr) > 0.5) ; signal 7.91 ppm with *p* (corr) = 0.45) (box on the right of horizontal axis). HCC-NAFLD is characterized by increased level of Glu/Gln (glutamate/glutamine) (signal 2.11–2.17 ppm and 2.45 ppm with *p* (corr) > 0.5) (top box on the vertical axis). These metabolites had VIP (Variable Influence on the Projection) > 1. NI: Not Identified.

**Figure 5:**
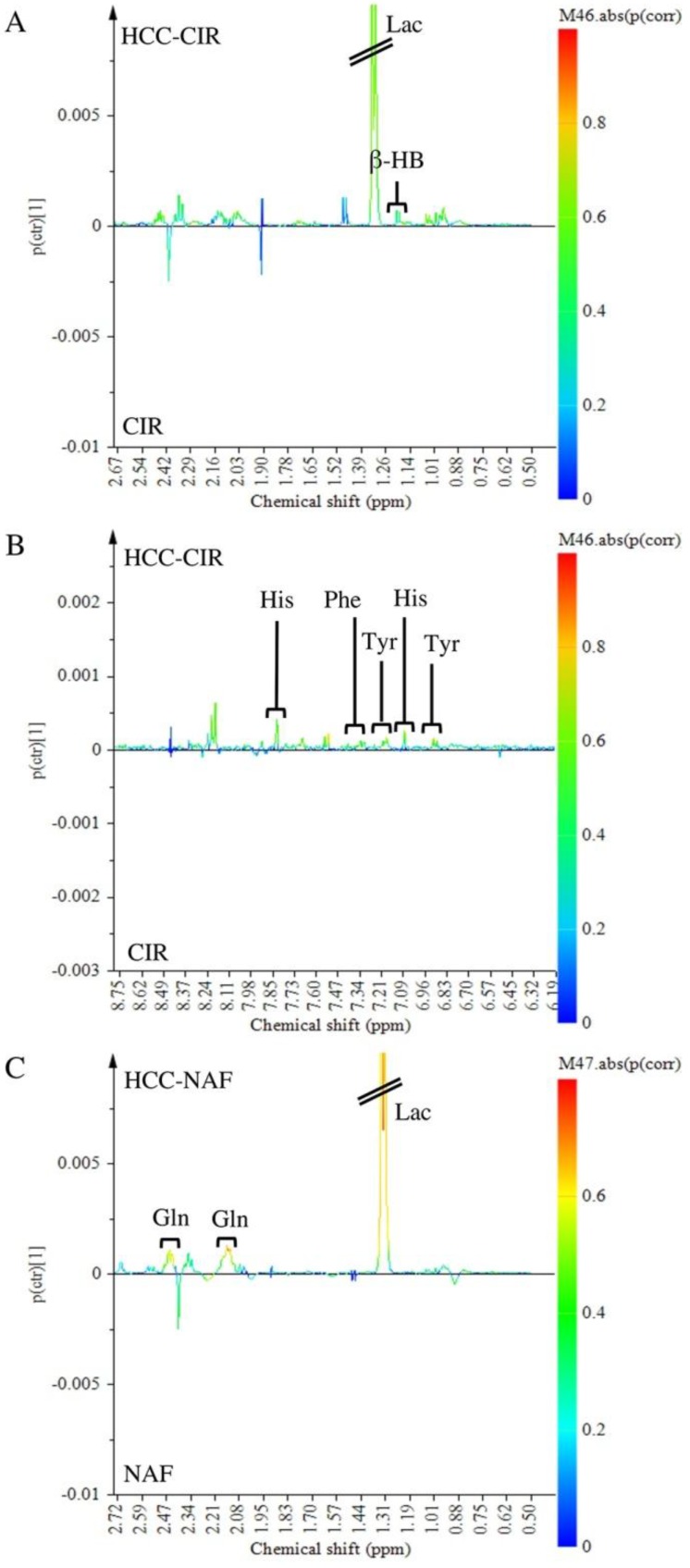
Focus on the loading S-line plot of Orthogonal Partial Least Square-Discriminant Analysis (OPLS-DA) discriminated HCC-CIR from Cirrhosis tissue (CIR) (**A**,**B**) (focus on Figure 3B) and OPLS-DA discriminated HCC-NAF from NAFLD tissue (NAFLD) (**C**) (focus on Figure 3D): variations of bucket intensities are represented using a line plot with a color scale for correlation coefficient.

The SUS-plot comparing the scaled loadings *p* (corr) of the two OPLS models, visualizes the shared metabolic contribution along the diagonal, and the unique metabolic contribution along the orthogonal axes (Figure 4). Common metabolic features to HCC-NAFLD and HCC-cirrhosis included the prominent high level of Lac (signals at 1.30–1.35 ppm) and low level of Glc (signals at 4.61–4.67; 5.20–5.28 ppm), displayed in the opposite corners of the plot. In contrast, metabolic specificities were displayed in boxes on horizontal and vertical axis: (i) in HCC-Cirrhosis: a high level of β-HB (signals at 1.18 ppm with *p* (corr) > 0.5 and signals at 1.19–1.20 ppm with *p* (corr) = 0.45) (Figure 5A), Tyr (signals at 3.94–3.95 ppm and 6.88–6.91 ppm with p (corr) > 0.5 ; signals at 7.17–7.18 ppm, with *p* (corr) = 0.45), Phe (signal 7.34–7.37 ppm with *p* (corr) > 0.5; signal 7.40–7.44 ppm with *p* (corr) = 0.45) and His (signal 7.07–7.09 ppm with *p* (corr) > 0.5); signal 7.91 ppm with *p* (corr) = 0.45) (Figure 5B); (ii) in HCC-NAFLD: high level of Glutamate (Glu) and Gln (signals at 2.11–2.17 ppm and 2.45 ppm with *p* (corr) > 0.5) (Figure 5C).

Taking together OPLS-DA models and SUS plots, comparing HCC-NAFLD and HCC-cirrhosis provide evidence that HCC-cirrhosis involve increased contribution of β-HB and aromatic amino acids (His, Tyr, Phe) and that HCC-NAFLD involve strong contribution of Gln/Glu.

### 2.5. Glutamine Synthetase Immunostaining

**Figure 6:**
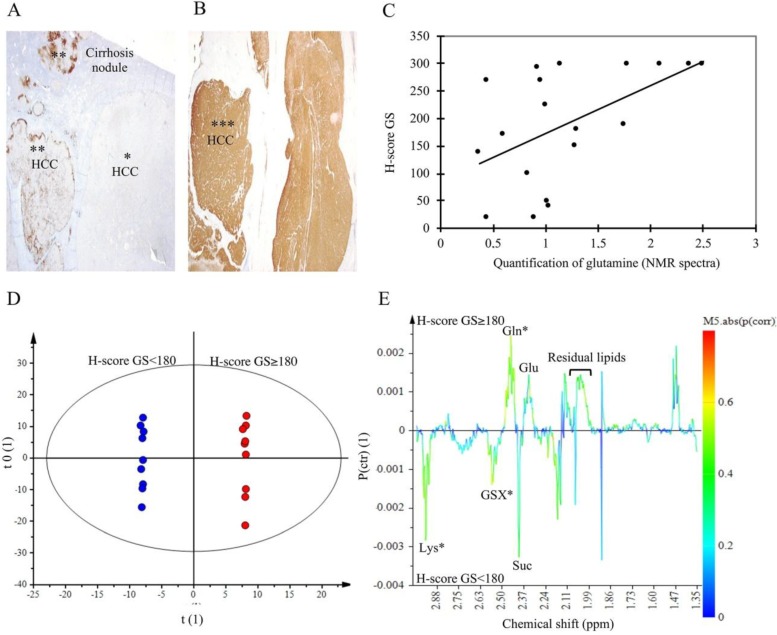
Glutamine Synthetase immuno-staining and correlation with NMR data. (**A**,**B**) Immunohistochemical expression of glutamine synthetase (GS): diffuse and intense staining with H-score = 300 (***); focal staining (**) and negative (*). X10. (**C**) correlation between quantification of glutamine on ^1^H-NMR spectra and GS H-Score, correlation coefficient = 0.53, *p* = 0.009 (test of Pearson). (**D**,**E**) Orthogonal Partial Least Square-Discriminant Analysis (OPLS-DA) score scatter plot and loading S-line plot of aqueous extract spectral data from 1.34 to 3 ppm of HCC with H-score < 180 (blue) versus HCC with H-score ≥ 180 (red). *n* = 18. The model was built with 1 predictive and 6 Y-orthogonal components and exhibited an explained variance: (R2X) of 0.69, (R2Y) of 1, predictability (Q2Y) of 0.79. The buckets are displayed according to the colored scale of correlation coefficient *p* (corr) (*: *p* (corr) > |0.5|).

To validate the increase of Gln in HCC-NAFLD, we analyzed Glutamine Synthetase (GS) expression by immunohistochemical staining on tumor tissues. GS immunostaining was realized on HCC-NAFLD tissues (*n* = 11), and HCC-Cirrhosis (*n* = 7) (Figure 6A,B). Intense immunoreactivity, defined by a H-score ≥ 180, the median value, concerned mainly HCC-NAFLD (63%) versus HCC-Cirrhosis (28%). In addition, in HCC-NAFLD, 34% had a GS staining H-Score equal to 300. This observation is consistent with our metabolomics data, since Gln quantification was correlated with GS staining H-score (Figure 6C). OPLS-DA focusing on NMR spectra from 1.34 to 3 ppm shows a strong discrimination between HCC with a GS H-score ≥ 180 and HCC with a GS H-score < 180 (Figure 6E). Among signals contributing to the discrimination (*p* (corr) > 050), we identified and confirmed Gln (2.45 ppm) in HCC exhibiting a high GS staining whereas GSX (2.55 ppm) and lysine (2.96 ppm) were found in HCC with a weak GS staining (Figure 6D). This data is consistent with the fact that lysine is a cetogenic amino acid involved in beta-oxydation of fatty acids; this metabolite might explain the overexpression of β-HB found in HCC developed on cirrhosis.

However, we did not find a significant correlation between GS expression (H-score) and stage of differentiation (well or moderately) (R2 = 0.44, *p*-value = 0.070, Spearman test) (data not shown).

## 3. Discussion

### 3.1. Advantage and Limitation of Using Tissue Extract Instead of Intact Tissue in this NMR Metabolomics Study

We performed our metabolic analysis on tissue extracts. Some NMR-based metabolomic studies have used intact tissue and magic angle spinning techniques[14,15]. In these studies, both lipid and water-soluble metabolites can be obtained in the same spectrum, and spectra are suited for comparison with MR spectral imaging *in vivo*. In comparison, NMR analysis of tissue extracts requires sample processing, separate analysis of lipid and water-soluble extracts, but has the important advantage to have improved spectral resolution, thus allowing detect more metabolites, especially those with a low concentration including succinate, fumarate, histidine, uracil, NAD in water-soluble extracts, arachidonic acid, linoleic acid in lipid extracts. In this study, we privileged spectral resolution and the number of detectable metabolites.

### 3.2. HCC Metabolic Profile Involves High Glycolysis and Impaired Phospholipids Metabolism

Few studies have investigated the metabolome of human HCC tissue by NMR-spectroscopy. Most studies of HCC have used Mass Spectrometry [16,17,18]. Our metabolomics approach combined with multivariate analysis reveals up-regulated levels of Lac, PC, PE, Gln, and Val and down-regulated levels of Glc and MUFA in HCC tissue in comparison with corresponding NTT. High level of Lac with low level of Glc confirms the well-known glycolytic shift found in cancer tissues, including human and animal model of HCC [14,19,20]. This features the “Warburg effect”, where high lactate production is observed even in the presence of oxygen and a low level of aerobic oxidation through the TCA cycle [21]. As previously reported for human HCC tissue, our data showed a decrease of MUFA content and a modification of phospholipids metabolism with an increased level of phospholipid derivatives, suggested a need for synthesis of membranes [14,16].

### 3.3. Metabolomics Shows Differences between HCC-NAFLD and HCC-Cirrhosis

To our knowledge, this study is the first to investigate tissue metabolome of human HCC according to the underlying pathology including histologically proven NAFLD. In contrast to others tumors (prostate, esophagus, breast etc.), HCC has the particularity to develop on underlying disease such as cirrhosis or NAFLD. As a way to “remove” the impact of the underlying liver disease on the metabolic profile of HCC, a SUS-plot analysis was performed, which allows to highlight common and specific metabolites of two groups of HCC associated with cirrhosis or with non-cirrhotic NAFLD.

This approach has revealed specific metabolites for each HCC, as an increased level of β-HB and aromatic amino acids in HCC-Cirrhosis and overexpression of Gln/Glu in HCC-NAFLD.

β-HB constitutes 70% of ketone bodies and is produced by liver mitochondria from fatty acid oxidation. β-HB was also reported to be increased in an animal model of HCC tissue [22]. Aromatic amino acids, Phe and Tyr, are oxidized into the TCA cycle after conversion into fumarate. Therefore, their accumulation in HCC-cirrhosis may indicate the impairment of the TCA cycle. Phe and Tyr were also found increased in viral B HCC tissue [16] and in serum of cirrhotic patients versus healthy controls [10]. An imbalance with decreased levels of branched-chain amino acids (BCAAs) and increased levels of aromatic amino acids (Tyr, Phe) is commonly seen in plasma of patients with advanced cirrhosis [23]. A recent study reported an association between the ratio of serum BCAAs/aromatic amino acids and HCC risk [24]. In addition to β-HB, accumulation in aromatic amino acids levels suggests impairment of mitochondrial function and inflammatory status in HCC-cirrhosis.

In our cohort, the group of HCC on non-cirrhotic tissue mostly included NAFLD. In particular, there were as many HCC developed on steatosis without inflammatory damages than HCC developed on NASH. Alexander et al. studied a large cohort of 157 HCC patients with non-cirrhotic fatty liver disease and pointed out that only 15% of underlying tissue show steatohepatitis, raising the hypothesis that HCC in NAFLD may arise in the absence of histologically evident inflammation [25]. It becomes necessary to identify metabolic specificities of HCC developed on NAFLD in absence of cirrhosis. The main result of this study is that HCC associated with non-cirrhotic NAFLD specifically exhibited a high level of Gln. This latter finding sustains the observations reported by Martinez-Granados et al., showing by tissue NMR that Gln levels were higher in non-cirrhotic tissue than in cirrhotic tissue [15]. In hepatocytes, Gln is implicated in ammonium detoxification, thus increased levels of Gln may reflect preservation of ammonium detoxification ability in non-cirrhotic tissue. Moreover, in tumor, Gln is a required substrate for biosynthesis and energy metabolism. Gln is a nitrogen donor for the *de novo* synthesis of both purines and pyrimidines, and is essential for production of nucleotides during cell proliferation. Oxidation of Gln carbon backbone in mitochondria is a major source of energy for proliferating cells, including tumor cell lines [26]. Gln is produced from Glu by glutamine synthetase (GS). The involvement of GS in liver tissue is wildly described associated with others (Glypican 3, heat shock protein 70) as biomarkers of hepatocellular tumors, particularly overexpressed in malignant tumors [27]. Indeed, Long et al., reported overexpression of GS in 70% of HCC versus 46.7% in cirrhosis and 38% in week fibrosis tissue, suggesting the role of GS in tumorigenesis and malignant progression [28]. Wasfy et al., also pointed out overexpression of GS in HCC and showed specificity and sensitivity of GS of 80% and 84% respectively [29]. The overexpression of GS is highly correlated with β-catenin mutation, and GS is proposed as a reliable marker of β-catenin activation secondary to its mutation [30]. Interestingly, low fibrosis was reported as an intriguing hallmark of β-catenin mutated tumors [30,31]. Our study is the first one to suggest a relationship between GS immunostaining and metabolic composition explored by NMR-spectroscopy. This observation might indicate the interest of the use *in vivo* of spectroscopy combined to MRI to detect hepatic lesions potentially β-catenin mutated. In our cohort, 63% of HCC-NAFLD has a diffuse GS immunostaining, among which 57% have a H-score equal to 300, versus 28% of HCC-cirrhosis. There are few histological data on GS labelling of HCC-NAFLD and actually, results from studies are conflicting [32,33].

## 4. Patients and Methods

### 4.1. Patients and Collection of Specimens

Liver tissue specimens were collected from 28 patients undergoing hepatectomy at the University Hospital of Clermont-Ferrand since November 2011 to May 2014 (*n* = 21) and from the tumor bank of the University Hospital of Saint-Etienne (*n* = 7). Written informed consent was obtained from each patient.

HCC was diagnosed before surgery using either radiological Barcelona criteria or liver biopsy histological analysis [34]. Diagnosis was established after surgery using pathological analysis. We did not include patients with a history of a second cancer for the last 2 years or who have received prior treatment for their HCC like chemoembolization. For each patient, tumor tissue (TT) and corresponding distant Non-Tumoral Tissue (NTT) (>2 cm) were collected. Specimen were transported in ice. The pathologists process to sampling on iced laboratory work plan. All tissue samples were snap frozen in liquid nitrogen to immediately “quench” metabolism, and stored at −80 °C until extraction to prevent metabolite destruction.

The study was approved by the Ethic Committee Sud-Est VI Clermont-Ferrand (Agreement number AU887, 04/03/2011).

### 4.2. Histology

Tissues were fixed in 10% formalin and embedded in paraffin for light microscopy. Paraffin sections with a thickness of 5 μm were stained with hematoxylin and eosin method. HCC type and grade of differentiation (WHO Classification) were established. NTT was characterized by the presence or not of lesions of chronic hepatitis, fibrosis, steatosis, steato-hepatitis and the METAVIR Score. Immunohistochemical analysis of Glutamine Synthetase (GS) was done according to standard procedures (Antibodies from BD Transduction Laboratories™, dilution at 1/400). To evaluate GS staining, a H-score (0–300) was calculated by multiplying staining intensity (0, negative; 1, weak; 2, moderate; 3, strong) with the positively stained area (0–100%) [32]. Immunohistochemical staining was performed on 24 samples (HCC-NAFLD *n* = 11, HCC-Cirrhosis *n* = 7, HCC-Normal *n* = 6).

### 4.3. Sample Preparation for NMR-Spectroscopy

Tissue samples were processed to obtain aqueous and lipid extracts. A piece of tissue (250 mg) was mixed with cold acetonitrile/water (1:1, v:v, 1.75 mL) and then homogenized, over ice, using a polytron. Samples were centrifuged (17,000 g, 20 min, 4 °C) and the aqueous supernatant was centrifuged (17,000 g , 15 min, 4°C) twice and dried to obtain the water-soluble fraction of liver extracts. The organic phase was dissolved in cold chloroform/methanol (2:1, v:v, 1.5 mL), homogenized, centrifuged (17,000 g, 20 min, 4 °C), then, filtered and dried to obtain the lipid phase. All reagents are conserved at 4 °C and all experiments are conducted in ice. All dried samples were stored at −20 °C.

### 4.4. ^1^H-NMR Spectroscopy

Spectroscopy was performed at room temperature using a Brucker Advance 400 spectrometer operating at 400.13 MHz. The dried extracts were rehydrated in 500 μL of D_2_O containing Phosphate Buffer (1%) (Aqueous extract) or 500 µL chloroform-d/methanol-d (2:1, v:v) (lipid extract) in 5 mm diameter NMR tubes. For all samples, a one-dimensional ^1^H-NMR spectrum was acquired using a ZGPRESAT sequence with water signal suppression at low power and the following parameters: 8 µs −90° pulse length, 10 s relaxation delay, 10 ppm spectral width, 128 transients and 32 K complex data points. Resonance assignment was carried out according to chemical shift values reported in the literature [35] and the free access database Human Metabolome Database (HMDB). We performed ^1^H-^1^H COSY and ^1^H-^13^C HSQC 2 dimensions NMR experiments in a few samples to definitely assign signals corresponding to phosphoethanolamine (PE) and succinate (Suc).

### 4.5. Data Processing

A line broadening factor of 0.3 Hz was applied to Free Induction Decay (FID) before Fourier transformation. ^1^H-NMR spectra were manually corrected for phase and baseline using MestReNova (Mestrelab Research chemistry software solutions). Peak referencing was done on the signal of creatine at 3.035 ppm for aqueous extracts and phosphatidylcholine (Ptcho) at 3.26 ppm for lipid extracts. The spectra were binned into 0.02 ppm width data samples (from 0 to 9 ppm for the aqueous phase spectra and from 0.2 to 4.4 ppm for the lipid phase spectra), then normalized to the total area under spectrum, after removing spectral regions containing solvent (water, methanol, chloroform) resonances.

### 4.6. Metabolites Quantification

Metabolite signals were quantified in all tissue spectra. For quantitative metabolomics profiling, spectra were imported into MestReNova and assigned signals were integrated. Quantification was performed by relating metabolite signals to a residual protein signal (lysine at 2.99 ppm), according to a technique derived from [13]. The Wilcoxon test was used to compare NTT and tumour tissues. In the comparison of metabolites levels between the groups of HCC tissue, Kruskal–Wallis test with Dunn’s correction was used (XLSTAT.V 2014.5.02, Addinsoft).

### 4.7. Multivariate Data Analysis

Spectral data were imported from Excel into SIMCA (SIMCA 13.0 Umetrics, Sweden) and preprocessed using Unit Variance scaling. Unsupervised (Principal Components Analysis, PCA) and supervised multivariate statistical analysis were performed (SIMCA). An Orthogonal Projection to Latent Structure-Discriminant Analysis (OPLS-DA) was run to discriminate groups. The model performance was assessed by parameters R2X, R2Y, and Q2 related to the explained variance of data, the predicted variance and the 7-fold cross validated predicted variance, respectively. For each analysis, a score plot is presented with the predictive (horizontal axis) and the first orthogonal component of the model (vertical axis). Each point in the score plot represented the projection of an NMR spectrum and thus a patient’s sample. The loading plot represented the covariance between the Y-response matrix and the signal intensity of the various spectral domains. The S-plot was used to visualize both the covariance (*p* (1)) and the correlation *p* (corr) (1) structure between the X-variables and the predictive score *t* (1). The S-line plot was applied to NMR spectral data. It visualized the *p* (ctr) (1) loading colored according to the absolute value of the correlation *p* (corr) (1). In addition, the difficulty to define a specific metabolic tissue profile of the HCC is due to the fact that HCC develop on a pre-existing pathologic liver having its own metabolic dysfunction. In this study, we proposed a multivariate statistic method (Shared and Unique Structure-plot), as a way to “remove” the underlying liver disease metabolic profile [36]. The SUS-plot consists in the projection of the scaled loadings *p* (corr) (1) from two OPLS models and visualizes the shared metabolic contribution along the diagonals, and the unique metabolic features along the respective axes, delineated by boxes in figures. Partial correlation coefficients (*p* (corr) (1)) ≥ 0.45 were considered significant.

## 5. Conclusions

Our metabolomics analysis allows to discriminate HCC associated with non-cirrhotic NAFLD from HCC associated with cirrhosis. These results should be considered as a preliminary study and these findings should be confirmed in a larger cohort. The observed unique metabolic alterations may be part of the specific carcinogenic processes in cirrhosis or non-cirrhotic NAFLD, and could be checked as candidate biomarkers of malignant transformation. These first results may be a draft of the metabolic background to epidemiological findings of non-cirrhotic NAFLD as a pre-cancerous liver disease.

## Figures and Tables

**Table 1:** Clinical, biological and pathological characteristics of 2 groups of patients.

Parameter		HCC-Cirrhosis	HCC-NAFLD	*p*-Value
**Number of patients**	-	9	13	-
**Sex**	Male	8 (89%)	11 (85%)	0.77
**Sex**	Female	1 (11%)	2 (15%)	0.77
	Median [IQR]	68 [64; 69]	67 [65; 71]	0.84
	Hepatitis B and C Viruses	2 (22%)	-	-
**Etiology**	Alcohol	0 (0%)	-	-
**Etiology**	Metabolic Syndrome	3 (33%)	-	-
	Metabolic Syndrome + Alcohol	4 (45%)	-	-
	Yes	7 (78%)	10 (77%)	0.96
	Waist Circumference > 94 cm (M) > 80 cm (F)	8 (89%)	9 (69%)	0.28
Metabolic Syndrome * (IDF/AHA/NHLBI 2009)	Triglycerides > 1.5 g/L or treatment	2 (22%)	3 (23%)	0.96
Metabolic Syndrome * (IDF/AHA/NHLBI 2009)	HDL-cholesterol < 0.4 g/L (M) < 0.5 g/L(F)	5 (56%)	7 (54%)	0.94
	Blood Pressure > 135/80 mmHg or hypertension treatment	5 (56%)	10 (77%)	0.29
	Fasting Glucose > 1 g/L or diabetes treatment	8 (89%)	9 (69%)	0.28
	18.5–24.9	1 (11%)	1 (8%)	0.78
	25–29.9	5 (56%)	7 (54%)	0.94
	30–34.9	3 (33%)	5 (38%)	0.81
**Body Mass Index**	35–40	0 (0%)	0 (0%)	-
**Body Mass Index**	<20 ng/mL	4 (45%)	11 (85%)	0.047
	20–200 ng/mL	3 (33%)	2 (15%)	0.96
	>200 ng/mL	2 (22%)	0	0.075
	Well	2 (22%)	5 (38%)	0.42
**Serum AFP level**	Moderately	7 (78%)	7 (54%)	0.25
**Serum AFP level**	Poorly	0 (0%)	1 (8%)	0.39
	**Differentiation (WHO Classification)**	-	-	-	-

Significant differences: *p* < 0.05 (Fisher test or Mann–Whitney test). * Metabolic syndrome: No Data for 3 patients. AFP: Alpha foeto protein; HDL: high density lipoprotein cholesterol.

**Table 2:** Metabolites quantification, median variation in HCC tissue compared to NTT. Significant differences *p* < 0.005 (Wilcoxon Test).

	Metabolic Subset	Range of Integrated Signal (ppm)	Abbreviation	Metabolite	Median Change [IQR] HCC/NTT %	*p*-Value
AQUEOUS PHASE	Glycolysis/TCA Cycle Derivatives	1.30–1.35	Lac	Lactate	49 [19; 113]	<0.0001
		1.43–1.49	Ala	Alanine	11 [−18; 50]	0.49
		1.90–1.93	Ace	Acetate	26 [−7; 135]	0.025
		2.38–2.41	Suc	Succinic Acid	−29 [−63; −6]	0.001
		4.61–4.67	Alpha-Glc	α-glucose	−48 [−71; −25]	0.001
		5.20–5.28	Beta-Glc	β-glucose	−58 [−77; −23]	<0.0001
		5.38–5.43	Glycogen	Glycogen	−23 [−39; 41]	0.47
		6.49–6.53	Fum	Fumarate	−11 [−37; 78]	0.99
	Ketone bodies	1.18–1.21	β-HB	β-hydroxybutyrate	8 [−19; 158]	0.2
	Glutamine Derivatives	2.28–2.39	Glu	Glutamate	19 [−13; 170]	0.019
		2.41–2.51	Gln	Glutamine	70 [9; 112]	0.0001
	Amino Acids	0.93–0.97	Leu	Leucine	46 [15; 92]	0.007
		1.02–1.04	Val	Valine	52 [19; 102]	0.002
		7.16–7.20	Tyr	Tyrosine	50 [−2; 145]	0.007
		7.39–7.44	Phe	Phenylalanine	43 [−16; 85]	0.009
		7.07–7.11	His	Histidine	21 [−27; 155]	0.09
	Amino Acid Derivatives	2.98–3.07	Creat	Creatine	−18 [−51; 8]	0.052
		2.70–2.73	Sar	Sarcosine	27 [−2; 163]	0.011
		2.50–2.54	GSX	Total Glutathione	−17 [−54; 86]	0.8
		3.52–3.56	Gly	Glycine	−44 [−65; −18]	0.08
		8.43–8.46	For	Formate	27 [−12; 139]	0.071
	Nucleotides	5.73–5.83	Uracil	Uracil	138 [24; 381]	<0.0001
	Vitamines and Co-factors	4.49–4.53	Asc A	Ascorbic acid	16 [−16; 43]	0.047
		8.40–8.43	NAD	NAD	50 [−3; 86]	0.024
	Phospholipid Derivatives	3.18–3.20	Cho	Choline	36 [−9; 110]	0.013
		3.21–3.22	PC	Phosphocholine	59 [−12; 96]	0.002
		3.22–3.23	GPC	Glycerophosphocholine	0 [−26; 24]	0.598
		3.99–4.02	PE	Phosphoethanolamine	88 [−25; 284]	0.01
LIPID PHASE	Phospholipid Derivatives	3.06–3.09	MethylPtEth	Methyl-phosphatidylethanolamine	0 [0; 100]	0.19
		3.12–3.18	PtEth	Phosphatidylethanolamine	3 [−21; 33]	1
		3.22–3.29	PtCho+SM	Phosphatidylcholine + Sphingomyeline	13 [−4; 46]	0.06
	Cholesterol	0.70–0.75	T Chol CH3	Total cholesterol CH_3_	29 [−8; 116]	0.025
		1.03–1.05	E Chol	Esterified cholesterol	−14 [−42;15]	0.07
	Fatty Acids	0.86–0.96	T FA	Total fatty acids (terminal CH_3_)	1 [−3; 6]	0.2
		1.59–1.70	β-CH2 FA	Fatty acids (β-CH_2_)	−7 [−15; 2]	0.038
		2.32–2.41	α-CH2 FA	Fatty acids (α-CH_2_)	−3 [−11; 10]	0.82
	Saturated Fatty Acids	1.24–1.44	Sat FA	Saturated FA -(CH_2_)n-	−1 [−4; 4]	0.9
	Unsaturated Fatty Acids	2.02–2.12	MUFA	Allylic -CH_2_-CH=CH Mono-Unsaturated fatty acids	−13 [−27; −1]	0.002
		5.23–5.53	UFA	Olefinic -CH=CH- FA	−6 [−15; 6]	0.2
		1.70–1.74	Ara A	CH_2_ arachidonic acid and eicosapentaenoic acid	−66 [−76; −35]	0.0001
		2.77–2.83	Lin A	CH2 linoleic acid	−19 [−36; −7]	0.0001
		2.85–2.95	PUFA	Diallylic CH_2_ Polyunsaturated fatty acids =CH-CH_2_-CH=	4 [−17; 21]	0.71
	TAG	4.15–4.25	TAG	Triacylglycerol	−6 [−14; 6]	0.17
	Glycero PL	3.99–4.10	GPL	Glycerophospholipid backbone CH_2_-OP	−2 [−24; 30]	0.62

## Abbreviations

Ace	Acetate
AFP	Alpha-Foeto-Protein
Ara A	Arachidonic Acid
Asc A	Ascorbic acid
BCAAs	Branched-Chain Amino Acids
BMI	Body Mass Index
Beta-Glc	β-glucose
Cho	Choline
Creat	Creatine
E Chol	Esterified Cholesterol
FID	Free Induction Decay
For	Formate
Fum	Fumarate
Gln	Glutamine
Glu	Glutamate
Gly	Glycine
GPC	Glycerophosphocholine
GS	Glutamine Synthetase
HBV	Hepatitis B Virus
HCC	Hepatocellular Carcinoma
HCV	Hepatitis C Virus
HDL	High Density Lipoprotein
His	Histidine
HMDB	Human Metabolome Database
H-NMR	Hydrogen-Nuclear Magnetic Resonance
HR-MAS MR	High Resolution Magic Angle Spinning Magnetic Resonance
Lac	Lactate
Leu	Leucine
Lin A	Linoleic Acid
MUFA	Monounsaturated Fatty Acid
NAFLD	Non-Alcoholic Fatty Liver Disease
NASH	Non-Alcoholic Steato-Hepatitis
NTT	Non Tumoral Tissue
OPLS-DA	Orthogonal Partial Least Square-Discriminant Analysis
PC	Phosphocholine
PCA	Principal Components Analysis
PE	Phosphoethanolamine
Phe	Phenylalanine
PLS	Partial Least Square
ppm	part per million
Ptcho	phosphatidylcholine
PUFA	Polyunsaturated Fatty Acid
Sar	Sarcosine
SM	Sphingomyelin
Suc	Succinate
SUS-Plot	Shared and Unique Structures-Plot
T Chol	Total Cholesterol
T FA	Total Fatty Acid
TAG	Triacylglycerol
TCA cycle	Citric Acid Cycle
TNF	tumor necrosis factor
TT	Tumor Tissue
Tyr	Tyrosine
Val	Valine
VIP	Variable Influence on the Projection
WHO	World Health Organisation
β-HB	β-hydroxybutyrate
